# Cinnamic Acid Increases Lignin Production and Inhibits Soybean Root Growth

**DOI:** 10.1371/journal.pone.0069105

**Published:** 2013-07-26

**Authors:** Victor Hugo Salvador, Rogério Barbosa Lima, Wanderley Dantas dos Santos, Anderson Ricardo Soares, Paulo Alfredo Feitoza Böhm, Rogério Marchiosi, Maria de Lourdes Lucio Ferrarese, Osvaldo Ferrarese-Filho

**Affiliations:** Laboratory of Plant Biochemistry, University of Maringá, Maringá, Paraná, Brazil; Instituto de Biología Molecular y Celular de Plantas, Spain

## Abstract

Cinnamic acid is a known allelochemical that affects seed germination and plant root growth and therefore influences several metabolic processes. In the present work, we evaluated its effects on growth, indole-3-acetic acid (IAA) oxidase and cinnamate 4-hydroxylase (C4H) activities and lignin monomer composition in soybean (*Glycine max*) roots. The results revealed that exogenously applied cinnamic acid inhibited root growth and increased IAA oxidase and C4H activities. The allelochemical increased the total lignin content, thus altering the sum and ratios of the *p*-hydroxyphenyl (H), guaiacyl (G), and syringyl (S) lignin monomers. When applied alone or with cinnamic acid, piperonylic acid (PIP, a *quasi*-irreversible inhibitor of C4H) reduced C4H activity, lignin and the H, G, S monomer content compared to the cinnamic acid treatment. Taken together, these results indicate that exogenously applied cinnamic acid can be channeled into the phenylpropanoid pathway via the C4H reaction, resulting in an increase in H lignin. In conjunction with enhanced IAA oxidase activity, these metabolic responses lead to the stiffening of the cell wall and are followed by a reduction in soybean root growth.

## Introduction

Many higher plants release allelochemicals into the environment through root excretion or exudation, leaching, evaporation and decomposition of plant tissues/organs [1]. These compounds can accumulate in the soil, influencing (positively or negatively) the growth and development of other species. This process is termed allelopathy and is broadly defined as any chemically-mediated interaction among plants. It also involves the contact of allelochemicals with the rhizosphere or bulk soil, and can be absorbed by receptor plants and exert its influence [2]. Due to the multitude of potential molecular targets, the investigation of the mode of action of allelochemicals is a challenging endeavor [3].

Many secondary metabolites have been referred to as allelochemicals. They are commonly found in soils at concentrations between 0.01 and 0.1 mM, and they affect plant growth at concentrations of up to 10 mM [4,5]. One of these compounds is trans-cinnamic acid, which is a well-known allelochemical that affects seed germination and root growth [1,3]. In most studies, the effects of cinnamic acid have been related to its action on the plasma membrane and related processes, including the induction of oxidative stress [6], an increase in reactive oxygen species (ROS) levels [1], a disturbance in Ca^2+^ homeostasis [7], and a decrease in the net nitrate uptake and plasma membrane H^+^-ATPase activity [8]. However, in higher plants, the cell wall is one of the first tissues affected by stress signals, which are then transmitted to the cell interior and influence several processes [9].

Lignification, which is the metabolic process of sealing a plant cell wall by lignin deposition, occurs during the course of normal tissue development and is an important step during root growth. Lignin is one of the last products of phenylpropanoid metabolism in plants, and it plays a crucial role in a plant’s resistance to biotic and abiotic stresses [10]. In addition to its allelopathic action, cinnamic acid is the first metabolite of the phenylpropanoid pathway and is consequently a precursor for lignin and flavonoid biosynthesis [11–13]. In this metabolic pathway, the first step is the deamination of L-phenylalanine by phenylalanine ammonia-lyase (PAL) to form *t*-cinnamate, which is hydroxylated by cinnamate 4-hydroxylase (C4H) to generate *p*-coumarate. The next step is the esterification of coenzyme A by 4-coenzyme A ligase (4CL) to produce *p*-coumaryl-CoA. Subsequently, 4-hydroxycinnamoyl-CoA: quinate shikimate 4-hydroxycinnamoyltransferase (HCT), in conjunction with *p*-coumarate 3-hydroxylase (C3H), hydroxylates *p*-coumaryl-CoA to form caffeoyl-CoA. A methoxylation reaction catalyzed by caffeoyl-CoA *O*-methyltransferase (CCoAOMT) generates feruloyl-CoA from caffeoyl-CoA. By the sequential action of cinnamoyl-CoA reductase (CCR), ferulate 5-hydroxylase (F5H), caffeic acid 3-*O*-methyltransferase (COMT) and cinnamyl alcohol dehydrogenase (CAD), the CoA thioesters are converted into *p*-coumaryl, coniferyl and sinapyl alcohols. In the last step of the pathway, cell-wall-bound peroxidase (POD) catalyzes the oxidative polymerization of these three monolignols, giving rise to the *p*-hydroxyphenyl (H), guaiacyl (G) and syringyl (S) units of the lignin polymer [11,14].

We have demonstrated previously that hydroxycinnamic acids, including ferulic, *p*-coumaric and caffeic acids, impaired soybean root growth and are associated with premature lignification and changes in the lignin monomer composition [15–17]. Based on these findings, a model of action has been proposed that indicates that these allelochemicals can be channeled into the phenylpropanoid pathway, which, in turn, may increase the lignin monomer amount, thus solidifying the cell wall and inhibiting the root growth of soybean plants. With this in mind, we hypothesized that exogenously applied cinnamic acid could act in a similar manner. The primary objective of this work was to analyze the effects of cinnamic acid on growth, C4H activity, and lignin and its monomer composition in soybean roots. Because the plant hormone indole-3-acetic acid (IAA) is required for plant growth, the activity of IAA oxidase was also evaluated.

## Materials and Methods

### General procedures

Soybean (*Glycine max* L. Merr. cv. BRS-184) seeds were surface-sterilized with 2% sodium hypochlorite for 3 min, rinsed extensively with deionized water and dark-germinated at 25°C on two sheets of moistened filter paper. Twenty-five 3-day-old seedlings of uniform size were supported by an adjustable acrylic plate and dipped into a 10×16 cm glass container filled with 200 mL of half-strength Hoagland’s solution (pH 6.0), with or without 0.1 to 1.0 mM cinnamic acid. All nutrient solutions were buffered with a 67 mM potassium phosphate buffer to eliminate the effects of a very low pH. Additional experiments with 0.1 mM piperonylic acid (PIP) were conducted, as indicated in the figure legends. The containers were kept in a growth chamber for 24 h at 25°C, with a light/dark photoperiod of 12/12 h and a photon flux density of 280 µmol m^−2^ s^−1^. The roots were measured before incubation and at the end of the experiment, and the difference in the lengths was calculated for all of the samples. Where indicated, the fresh root weight was determined immediately after incubation, and the dry weight was estimated after oven-drying at 80°C until a constant weight. *trans-*Cinnamic acid, PIP and IAA were purchased from Sigma Aldrich (St Louis, MO, USA), and all other reagents used were of the purest grade available or of chromatographic grade.

### Enzymatic assays

Indole-3-acetic acid (IAA) oxidase (E.C. 1.11.1.8) was extracted as described by Talwar et al. [18] with modifications. Fresh roots (1.0 g) were ground at 4°C in 2 mL of 100 mM phosphate buffer (pH 6.0); the homogenate was centrifuged (18,400×g, 20 min) and the supernatant was used as the enzyme preparation. The reaction mixture contained 20 µg of IAA, 0.2 mM 2,4-dichlorophenol, 0.2 mM MnCl_2_ and a suitable amount of enzyme extract in a final volume of 1 mL. The mixture was incubated for 15 min in darkness, and the reaction was stopped with 50 µL of 5 M HCl. The samples were filtered through a 0.45-µm disposable syringe filter (Hamilton^®^ Co, Nevada, USA) and analyzed (20 µL) in a high performance liquid chromatography (HPLC) system (Shimadzu^®^ Prominence 20, Tokyo, Japan) with a quaternary pump, an auto-sampler, a column oven, and a diode-array detector. A reversed-phase Shimpack^®^ CLC-ODS column (150 mm × 4.6 mm, 5 µm) was used at 30°C with an equivalent pre-column. The mobile phase was methanol: acetic acid 4% (30:70, v/v), with a flow rate of 0.8 mL min^−1^ for an isocratic run of 30 min. UV detection was performed at 280 nm. IAA, i.e., the substrate of IAA oxidase, was identified by comparing its retention time with a standard compound. A parallel control without enzyme extract was performed, and IAA was identified in a similar manner. IAA oxidase activity is expressed as nmol IAA consumed min^−1^ g^−1^ fresh weight.

Cinnamate 4-hydroxylase (C4H; E.C. 1.14.13.11) was extracted as described by Enkhardt and Pommer [19]. Fresh roots (0.5 g) were ground at 4°C in 1 mL of extraction medium containing 100 mM phosphate buffer (pH 7.6), 10 mM potassium chloride, 1 mM magnesium chloride, 8 mM EDTA and 15 mM 2-mercaptoethanol. The homogenates were centrifuged at (10,000×g, 10 min), and the supernatants were used as the enzyme preparations. For the C4H activity assay, the reaction mixture contained 100 mM phosphate buffer (pH 7.6), 1.33 mM cinnamic acid, 2 mM NADPH and a suitable amount of enzyme extract in a final volume of 1.5 mL. The mixture was incubated at 35°C, and the reaction was stopped after 40 min by the addition of 75 µL of 5 M HCl. The samples were filtered through a 0.45-µm disposable syringe filter (Hamilton^®^ Co, Nevada, USA) and analyzed (20 µL) in an HPLC system (Shimadzu^®^ 10AVP, Tokyo, Japan) equipped with a pump, a Rheodyne^®^ manual sample injector, and an UV detector. A reversed-phase Shimpack^®^ CLC-ODS column (150 mm × 4.6 mm, 5 µm) was used at room temperature together with an equivalent pre-column. The mobile phase was 70% methanol (v/v), with a flow rate of 0.7 mL min^−1^ for an isocratic run of 15 min. UV detection was carried out at 275 nm. The substrate of C4H, *t*-cinnamate, was identified by comparing its retention time with a standard compound. C4H activity is expressed as nmol *t*-cinnamate consumed min^−1^ g^−1^ fresh weight.

### Quantification of lignin and monomer composition

After the incubation period, dry roots (0.3 g) were homogenized in 50 mM potassium phosphate buffer (7 mL, pH 7.0) with a mortar and pestle and transferred into a centrifuge tube [20]. The pellet was centrifuged (1,400×g, 4 min) and washed by successive stirring and centrifugation as follows: 2× with phosphate buffer pH 7.0 (7 mL); 3× with 1% (v/v) Triton^®^ X-100 in pH 7.0 buffer (7 mL); 2× with 1 M NaCl in pH 7.0 buffer (7 mL); 2× with distilled water (7 mL); and 2× with acetone (5 mL). The pellet was dried in an oven (60°C, 24 h) and cooled in a vacuum desiccator. The dry matter obtained was defined as a protein-free cell wall fraction. All dry protein-free tissue was placed into a screw-cap centrifuge tube containing the reaction mixture (1.2 mL of thioglycolic acid plus 6 mL of 2 M HCl) and heated (95°C, 4 h). After cooling at room temperature, the sample was centrifuged (1,400×g, 5 min), and the supernatant was discarded. The pellet contained the complex lignin–thioglycolic acid (LTGA). The pellet was washed 3× with distilled water (7 mL), and the LTGA was extracted by shaking (30°C, 18 h, 115 oscillations min^−1^) in 0.5 M NaOH (3 mL). After centrifugation (1,400×g, 5 min), the supernatant was stored. The pellet was washed again with 0.5 M NaOH (3 mL) and mixed with the supernatant obtained earlier. The combined alkali extracts were acidified with concentrated HCl (1.8 mL). After precipitation (0°C, 4 h), the LTGA was recovered by centrifugation (1,400×g, 5 min) and washed 2× with distilled water (7 mL). The pellet was dried at 60°C, dissolved in 0.5 M NaOH, and diluted to yield an appropriate absorbance for spectrophotometric determination at 280 nm. Lignin was expressed as mg LTGA g^−1^ dry weight.

The oxidation with alkaline nitrobenzene was used to determine the lignin monomer composition [17]. The protein-free cell wall fraction (50 mg) obtained above was sealed in a Pyrex^®^ ampule containing 1 mL of 2 M NaOH and 1 mL of nitrobenzene and heated to 170°C for 150 min, and the sample was occasionally shaken during the reaction. The sample was then cooled to room temperature, washed 2× with chloroform, acidified to pH 3-4 with 5 M HCl and extracted twice with chloroform. The organic extracts were combined, dried, resuspended in 1 mL of methanol and diluted in methanol/acetic acid 4% in water (20:80, v/v). All of the samples were filtered through a 0.45-µm disposable syringe filter and analyzed by HPLC. The mobile phase was methanol/acetic acid 4% in water (20:80, v/v), with a flow rate of 1.2 mL min^−1^ for an isocratic run of 20 min. Quantification of the monomer aldehyde products (*p*-hydroxybenzaldehyde, vanillin and syringaldehyde) released by the nitrobenzene oxidation was performed at 290 nm using the corresponding standards. The results are expressed as mg monomer g^−1^ cell wall.

### Statistical analysis

The experimental design was completely randomized, and each plot was represented by one glass container with 25 seedlings. The data were expressed as the mean of four to six independent experiments ± standard error. An analysis of variance to test the significance of differences was performed with the statistical program Prism® (GraphPad 5.0, USA) and Sisvar^®^ (UFLA, Brazil). The differences between the parameters were evaluated using Dunnett’s multiple comparison test (Prism® software) and the Scott-Knott test (Sisvar^®^ software). P values ≤ 0.05 were considered to be statistically significant.

## Results

In comparison with the control, the root length was reduced by 59.3% to 92.6% with concentrations of cinnamic acid increasing from 0.25 to 1.0 mM (Table 1). The effects of the allelochemical were also evident for root weight, which significantly decreased by 21.5% to 34.9% (fresh weight) and 10.4% to 22.7% (dry weight) after 0.5 to 1.0 mM exposures, when compared with the control.

**Table 1:** Changes in the root length and fresh and dry weights of soybean seedlings treated for 24 h with cinnamic acid.

Cinnamic acid (mM)	Root length (cm)	%	Fresh weight (g)	%	Dry weight (g)	%
0	2.16 ± 0.08		2.75 ± 0.05		0.163 ± 0.001	
0.1	1.96 ± 0.14		2.54 ± 0.01		0.160 ± 0.002	
0.25	0.88 ± 0.08^*^	-59.3	2.26 ± 0.03		0.157 ± 0.003	
0.5	0.43 ± 0.03^*^	-80.1	2.16 ± 0.02^*^	-21.5	0.146 ± 0.001^*^	-10.4
0.75	0.18 ± 0.01^*^	-91.7	2.17 ± 0.02^*^	-21.1	0.144 ± 0.003^*^	-11.7
1.0	0.16 ± 0.01^*^	-92.6	1.79 ± 0.04^*^	-34.9	0.126 ± 0.002^*^	-22.7

Values (n 4 ± SE) significantly smaller than the control (P ≤ 0.05, Dunnett’s multiple comparison test) are marked with asterisk (*) The symbol % represents inhibition of statistically significant means in comparison to the control (0 mM).

**Figure 1:**
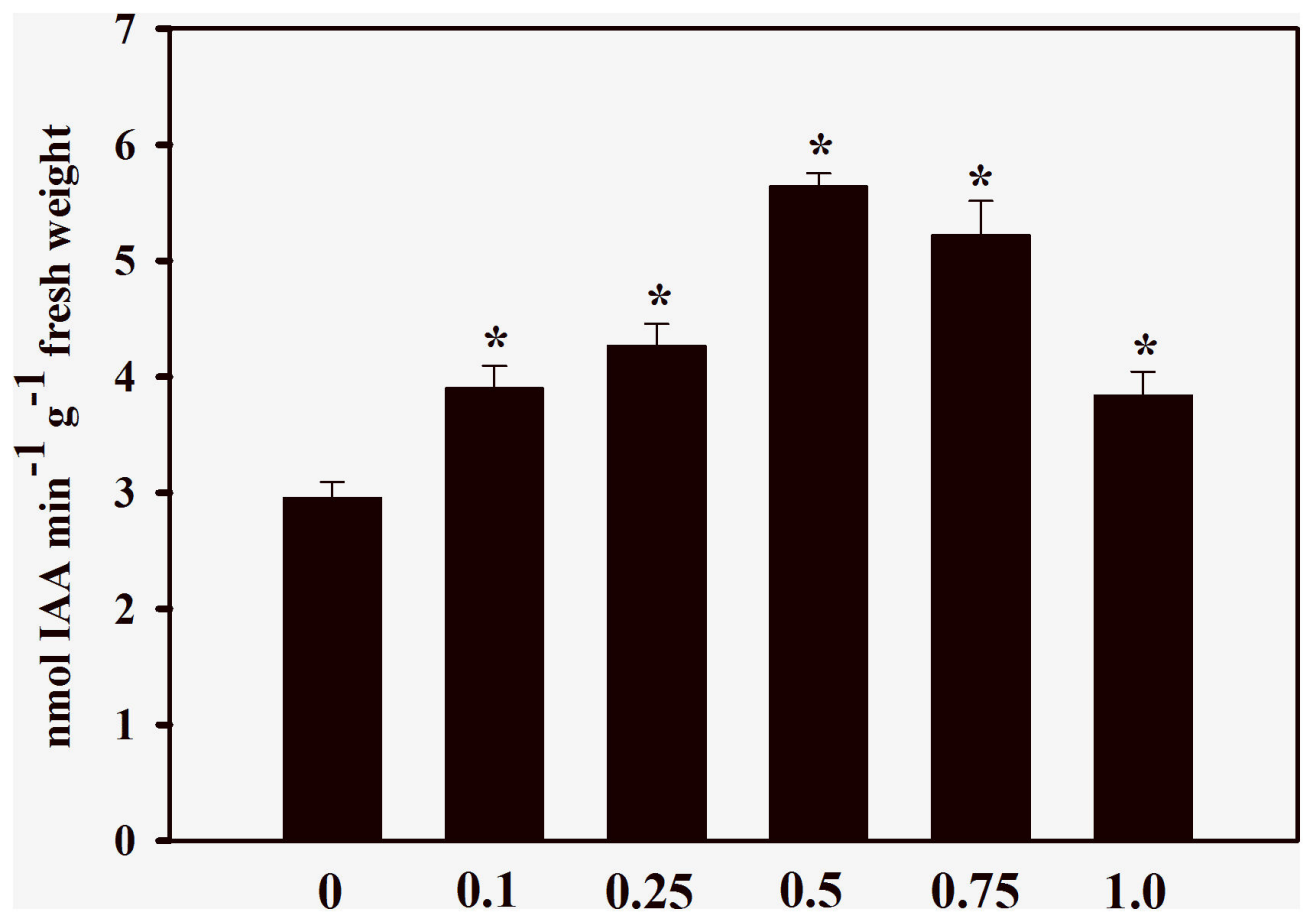
Effects of cinnamic acid on indole-3-acetic acid (IAA) oxidase activity. Values (n = 6 ± SE) significantly higher than the control (P ≤ 0.05, Dunnett’s multiple comparison test) are marked with *.

**Figure 2:**
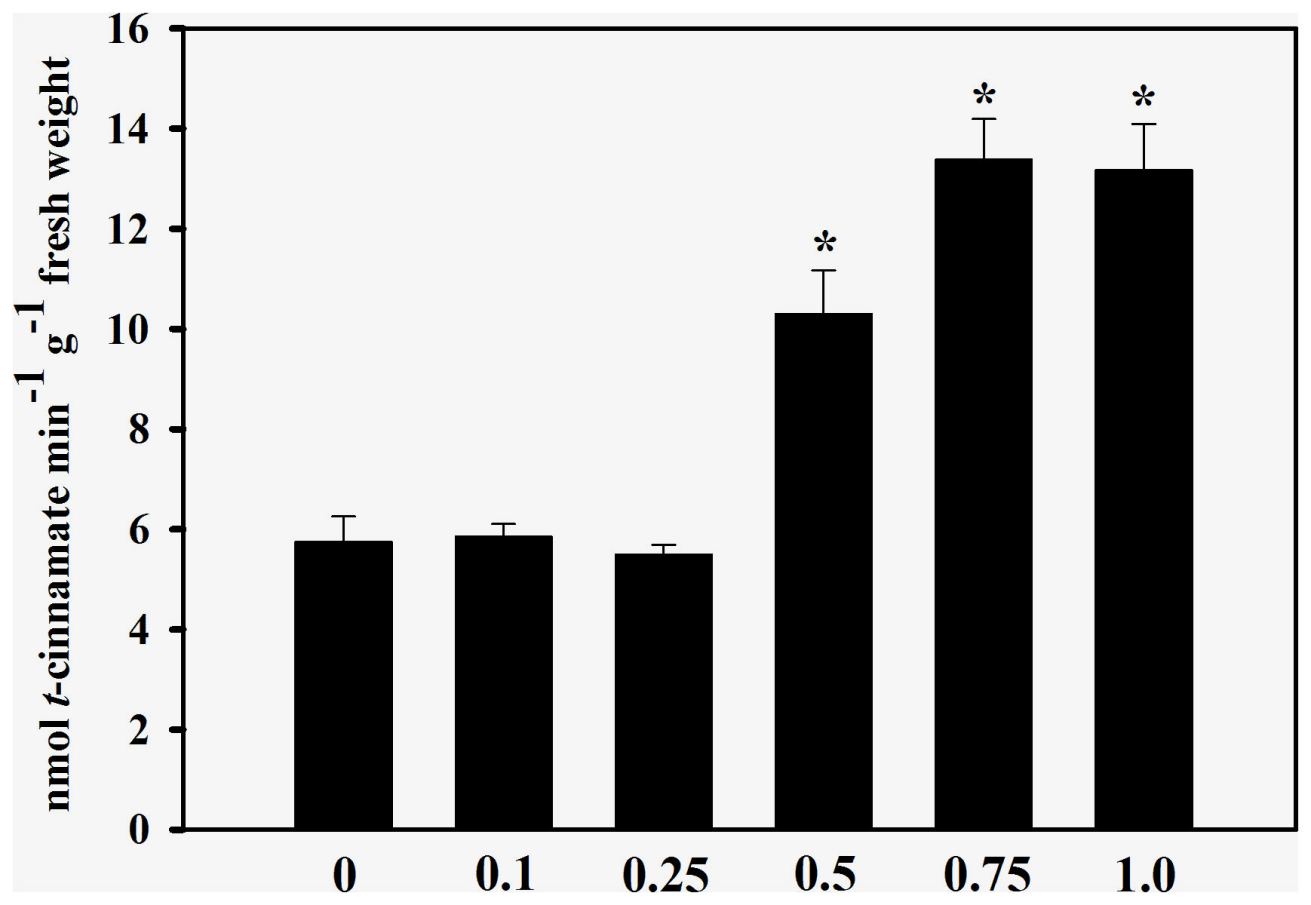
Effects of cinnamic acid on cinnamate 4-hydroxylase (C4H) activity. Values (n = 5 ± SE) significantly higher than the control (P ≤ 0.05, Dunnett’s multiple comparison test) are marked with *.

**Figure 3:**
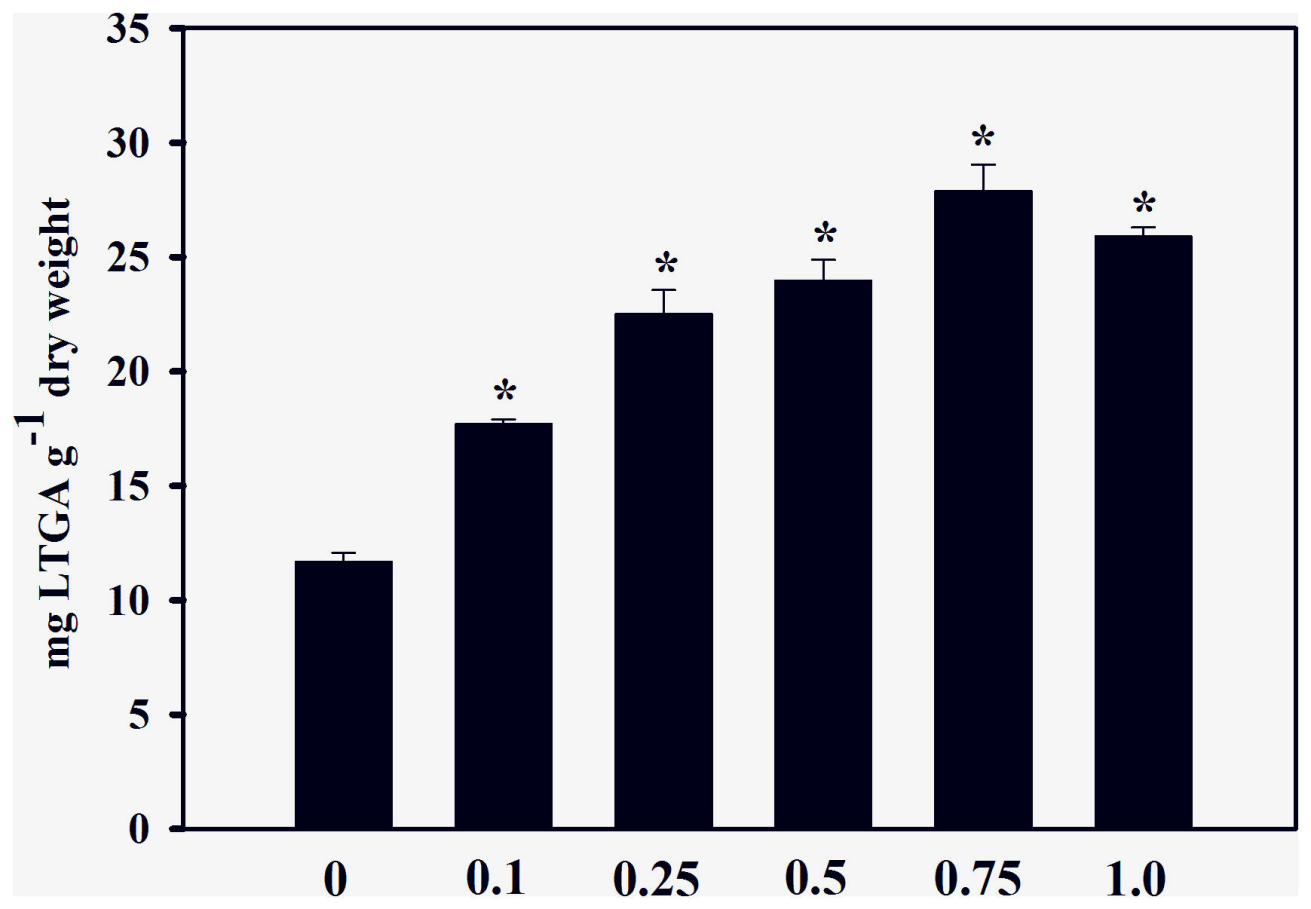
Effects of cinnamic acid on lignin content. Values (n = 4 ± SE) significantly higher than the control (P ≤ 0.05, Dunnett’s multiple comparison test) are marked with *.

In agreement with the effects observed on root growth, the enzyme activity of seedlings treated with cinnamic acid was also significantly different from those of controls. Roots exposed to cinnamic acid significantly increased IAA oxidase activity by 32% to 91% (0.1 to 0.5 mM); 76% (0.75 mM) and 30% (1.0 mM) in comparison with the control (2.96 ± 0.31 nmols min^−1^ g^−1^ fresh weight) (Figure 1). Cinnamic acid treatments also increased the C4H activities by 79% (0.5 mM) to 129% (≥ 0.75 mM) in comparison with the control (5.75 ± 0.51 nmols min^−1^ g^−1^ fresh weight) (Figure 2). The allelochemical increased lignin content by 51% to 138% after 0.1 to 0.75 mM treatments, in comparison with the control (11.7 ± 0.38 mg g^−1^ dry weight) (Figure 3). At a concentration of 1.0 mM, cinnamic acid increased the lignin content by 121% when compared with the control.

Having already ascertained that lignin content was affected by cinnamic acid, we investigated the lignin monomer composition by alkaline nitrobenzene oxidation (Table 2). This procedure degrades lignin, forming *p*-hydroxybenzaldehyde from *p*-hydroxyphenyl (H), vanillin from guaiacyl (G) and syringaldehyde from syringyl (S). Cinnamic acid treatments increased the H monomer content by 83%–417% when compared with that in the control (0.18 ± 0.01 mg g^−1^ dry weight). The G monomer content was not affected, but the allelochemical decreased the S monomer content by 21% (at 0.5 mM) and 29% (at 0.75 mM) in comparison with the control (0.34 ± 0.01 mg g^−1^ dry weight). The sum (H+G+S) of the lignin monomer content increased by 11%–46%, compared with that in untreated roots (1.86 ± 0.08 mg g^−1^ dry weight).

**Table 2:** Changes in the lignin monomer composition of soybean roots untreated (0 mM) or treated with cinnamic acid for 24 h.

Cinnamic acid (mM)						
Lignin monomer	0	0.1	0.25	0.5	0.75	1.0
H	0.18 ± 0.01	0.33 ± 0.02^*^	0.47 ± 0.03^*^	0.46 ± 0.02^*^	0.65 ± 0.04^*^	0.93 ± 0.06^*^
G	1.34 ± 0.06	1.42 ± 0.11	1.43 ± 0.11	1.39 ± 0.06	1.44 ± 0.12	1.47 ± 0.08
S	0.34 ± 0.01	0.32 ± 0.03	0.32 ± 0.01	0.27 ± 0.02^*^	0.24 ± 0,02^*^	0.31 ± 0.04
H + G + S	1.86 ± 0.08	2.07 ± 0.16^*^	2.22 ± 0.10^*^	2.12 ± 0.08^*^	2.33 ± 0.15^*^	2.71 ± 0.16^*^
H : G : S	10 : 72 : 18	16 : 69 : 15	21 : 65 : 14	22 : 65 : 13	28 : 62 : 10	34 : 54 : 12

H, p-hydroxyphenyl; G, guaiacyl; and S, syringyl monomers. Results are expressed as mg monomer g^− 1^ cell wall. Values (n = 5 ± SE) significantly different from the control (P ≤ 0.05, Dunnett’s multiple comparison test) are marked with asterisk (*)

**Figure 4:**
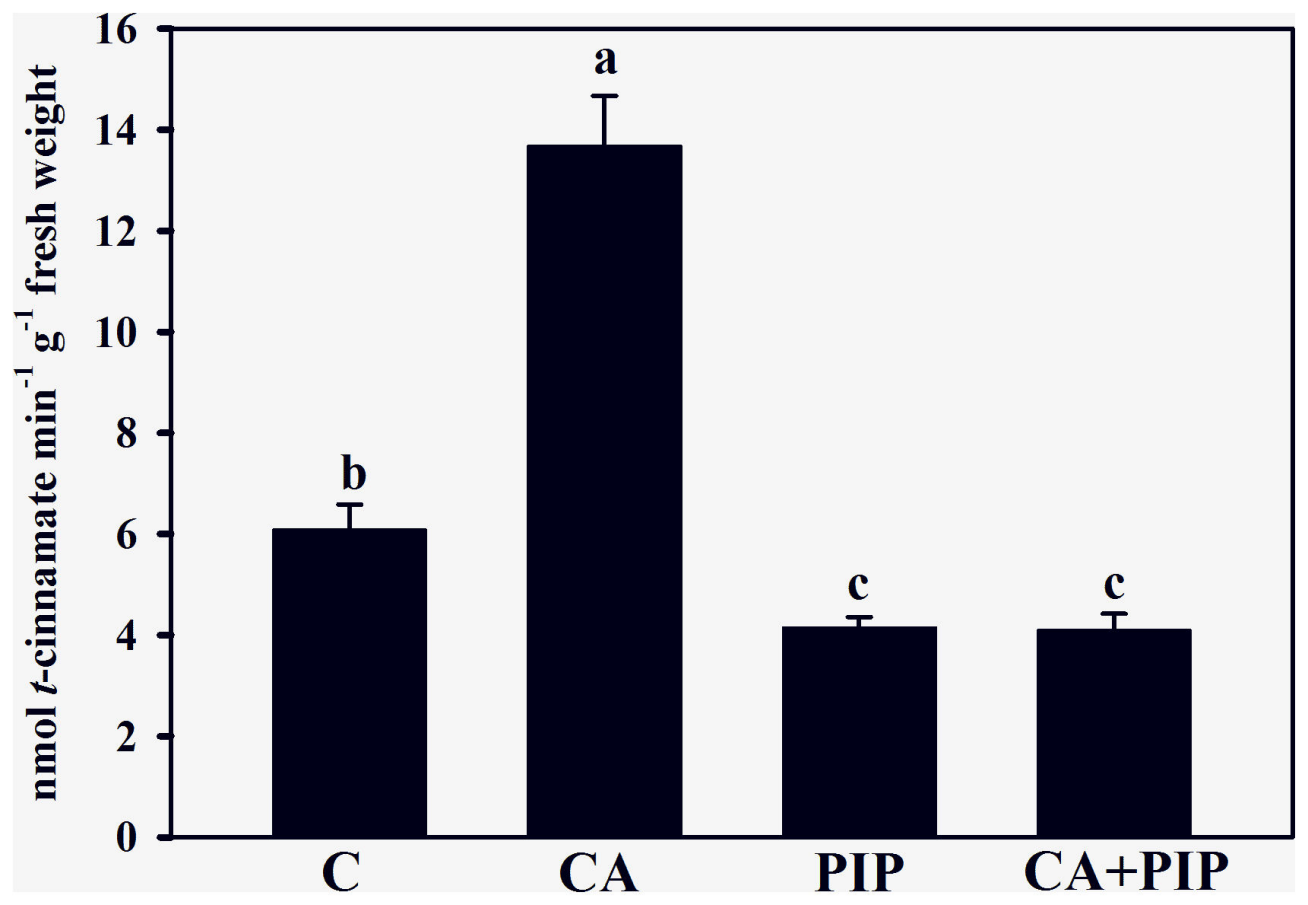
Effects of cinnamic acid on cinnamate 4-hydroxylase (C4H) oxidase activity of soybean roots untreated (C) or treated with 1.0 mM cinnamic acid (CA), 0.1 mM piperonylic acid (PIP) and 1.0 mM cinnamic acid plus 0.1 mM piperonylic acid (CA + PIP). Values (n = 4 ± SE) marked with different letters are significantly different (P ≤ 0.05, Scott-Knott test).

Hypothesizing that cinnamic acid can be channeled into the phenylpropanoid pathway via the C4H reaction, soybean roots were treated with PIP, which is a *quasi*-irreversible inhibitor of this enzyme. Figure 4 reveals that PIP reduced C4H activity by 32% (PIP alone) and 33% (cinnamic acid plus PIP) in comparison with the control (6.09 ± 0.49 nmols min^−1^ g^−1^ fresh weight). The treatment of roots with PIP alone or with the allelochemical plus PIP reduced the enzyme activity by 69% and 70%, respectively, in comparison with the cinnamic acid treatment (13.69 ± 0.99 nmols min^−1^ g^−1^ fresh weight).

**Figure 5:**
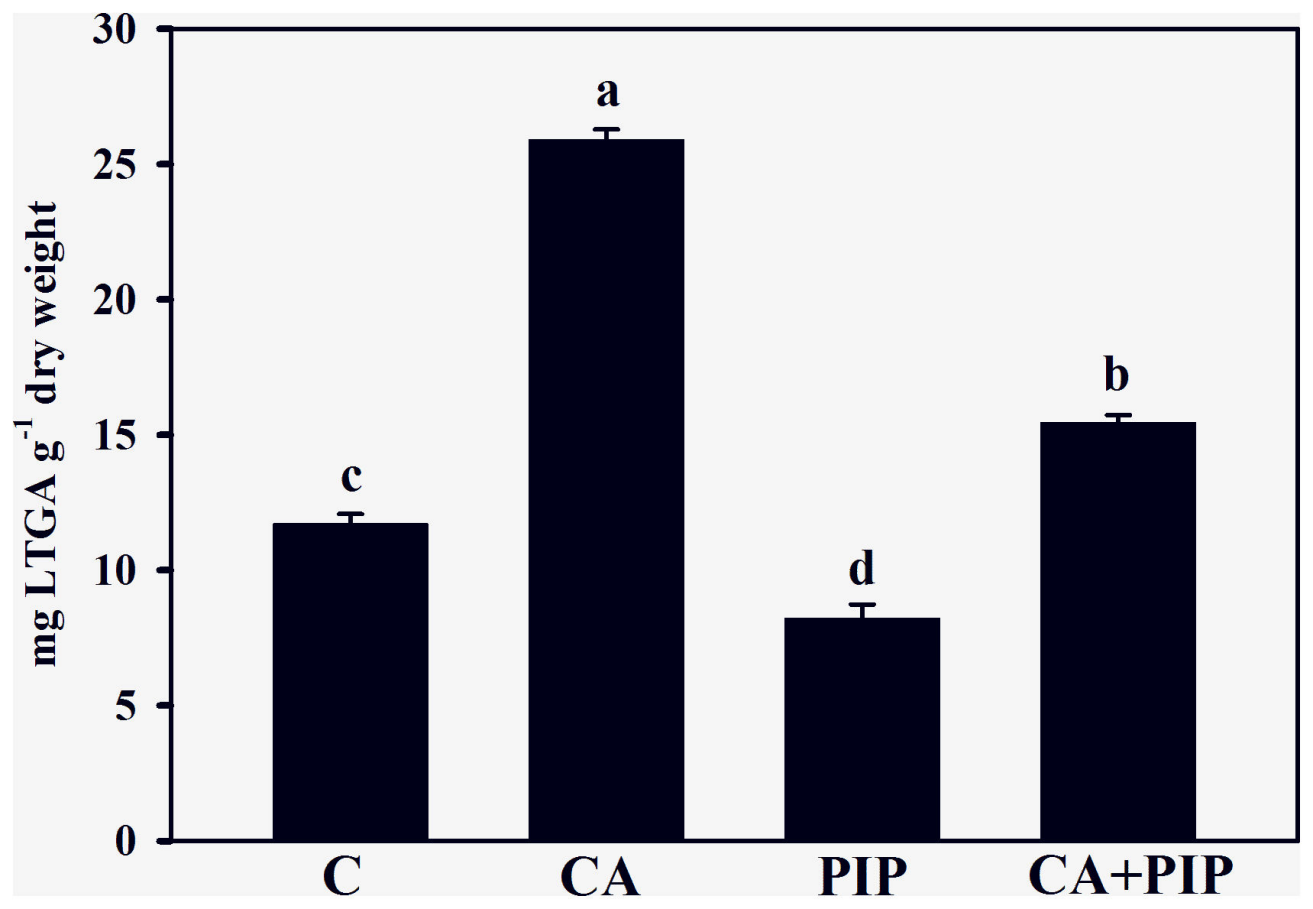
Effects of cinnamic acid on lignin content of soybean roots untreated (C) or treated with 1.0 mM cinnamic acid (CA), 0.1 mM piperonylic acid (PIP) and 1.0 mM cinnamic acid plus 0.1 mM piperonylic acid (CA + PIP). Values (n = 4 ± SE) marked with different letters are significantly different (P ≤ 0.05, Scott-Knott test).

**Figure 6:**
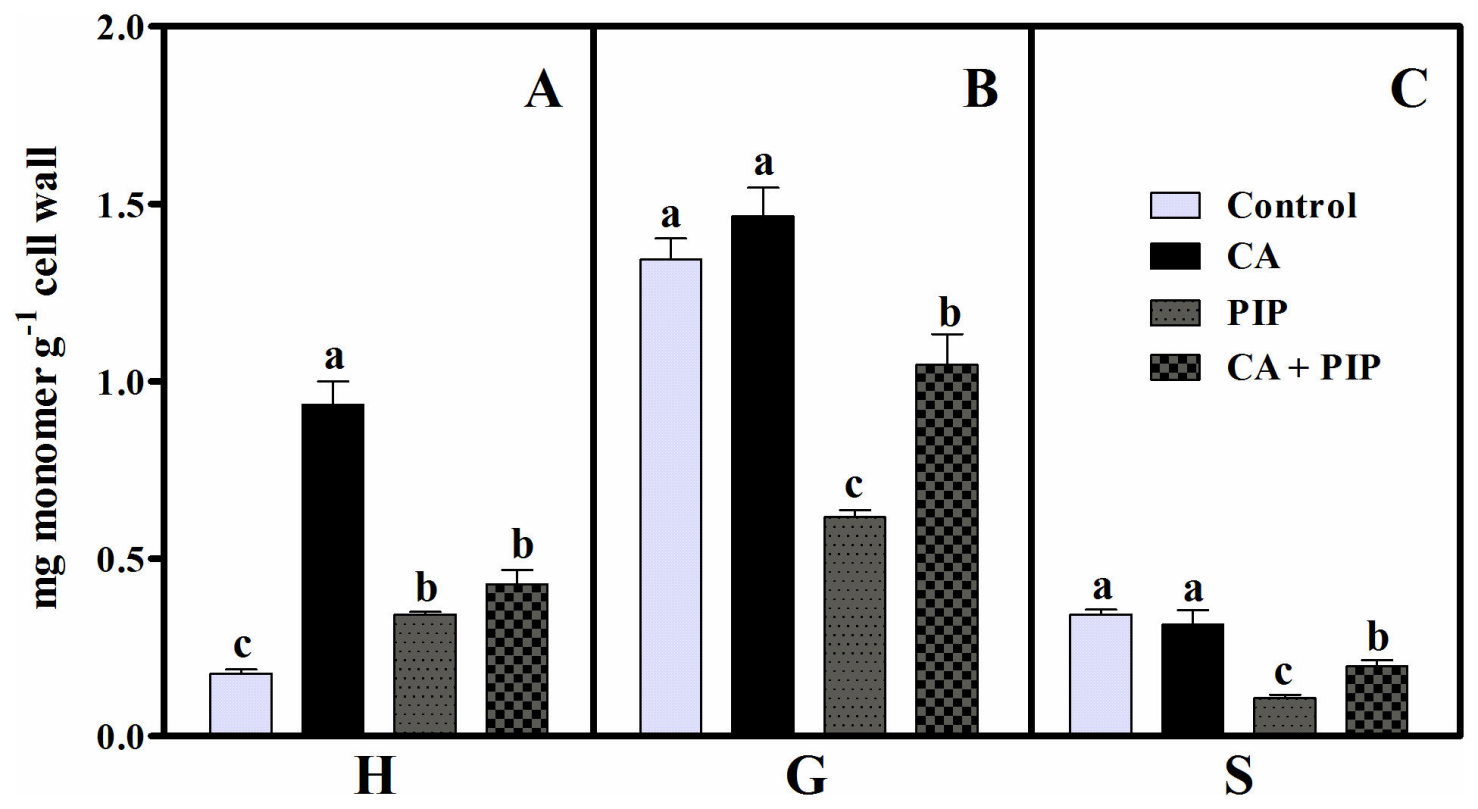
Effects of cinnamic acid on the lignin monomer composition of soybean roots untreated (C) or treated with 1.0 mM cinnamic acid (CA), 0.1 mM piperonylic acid (PIP) and 1.0 mM cinnamic acid plus 0.1 mM piperonylic acid (CA + PIP). H, *p*-hydroxyphenyl; G, guaiacyl; and S, syringyl monomers. Values (n = 4 ± SE) marked with different letters are significantly different (P ≤ 0.05, Scott-Knott test).

Because the inhibition of C4H by PIP can have a significant effect on lignin synthesis, subsequent analyses were conducted to evaluate the changes in lignin content in response to this inhibitor (Figure 5). The results indicate that PIP reduced the lignin content by 30% in comparison with the control (11.7 ± 0.38 mg^−1^ dry weight) and 68% in comparison with the cinnamic acid treatment (25.9 ± 0.40 mg^−1^ dry weight). Figure 5 also reveals that when used with cinnamic acid, the inhibitor (CA + PIP) reduced the lignin content by 40% in comparison with the cinnamic acid treatment. To determine whether the effects of PIP on lignin content could alter its monomer composition, further analysis was conducted on soybean roots (Figure 6). The data show that PIP reduced the three monomers of lignin in comparison with the cinnamic acid treatment (0.93 ± 0.06 mg^−1^ dry weight). The inhibitor reduced the H, G and S monomers by 63%, 58% and 66%, respectively. Finally, the exposure of roots to the allelochemical plus inhibitor (CA + PIP) reduced the H (54%), G (29%) and S (37%) monomers, in comparison with the cinnamic acid treatment alone.

## Discussion

Our findings showed that cinnamic acid (up to 1.0 mM) reduced soybean root growth and the fresh and dry weights (Table 1). It is known that plant roots are susceptible to allelochemicals in early seedling growth [21]. Of the many allelochemicals, cinnamic acid has proven to be responsible for strong inhibitory effects on root lengths and on fresh and dry weights of different plant species [1,3,7,8,22–25]. In soybean, Baziramakenga et al. [26] demonstrated that 0.05 to 0.25 mM cinnamic acid reduced the root and shoot dry biomass. Moreover, the growth of soybean seedlings and the formation of lateral roots were inhibited by this allelochemical, and the extent of inhibition was related to its concentration. Also in soybean, Ng et al. [27] observed that cinnamic acid at a concentration of 1.0 mM significantly reduced seedling fresh weight, seedling length and root length. These results are in agreement with studies conducted on cinnamic acid derivatives such as ferulic, *p*-coumaric and caffeic acids, which all inhibited the root growth (in terms of length and biomass) of soybean [16–28]. In brief, our findings confirm the role of cinnamic acid as a strong allelochemical. Nevertheless, little is known about its mode of action at the cellular level.

It is well established that IAA, which is the main auxin in higher plants, plays a vital role in early seedling growth and in responses to environmental stimuli. In general, IAA modulates processes such as tropic responses to light and gravity, general root and shoot architecture, organ patterning, vascular development and growth in tissue culture. Biologically active in its free form, IAA is inactivated immediately after, or concurrently with, a plant growth-promoting action. The rate of IAA degradation is closely associated with the enhanced activity of IAA oxidase [29,30]. As noted herein, significant increases in IAA oxidase activity in soybean roots occurred after cinnamic acid treatments (Figure 1). Previous reports provide evidence that supports these findings because cinnamic acid antagonizes the growth-promoting effect of IAA [31] and alters its biosynthesis [32]. Ferulic acid, which is a cinnamic acid derivative, reduced maize root length and increased the activity of IAA oxidase [33], while a decrease in IAA levels has been noted in maize stems treated with *p*-coumaric and ferulic acids [34]. In addition, Liu et al. [35] suggest that the activity of IAA oxidase can be increased by lignification during soybean hypocotyl growth. Because IAA is required for plant growth, the cinnamic acid-induced inhibition of soybean roots may be due to the decline of endogenous IAA caused by an increase in IAA oxidase activity (Figure 1). Future studies should be conducted to quantify the IAA levels in soybean roots exposed to cinnamic acid.

An interesting fact revealed in the current study is that the changes in root growth and IAA oxidase activities were accompanied by increases in C4H activity (Figure 2) and lignin content (Figure 3). C4H is the first P450-dependent monooxygenase of the phenylpropanoid pathway, and it is widely expressed in various tissues, particularly in roots and cells undergoing lignification [36]. In fact, Ye [37] demonstrated a correlation between C4H activity and the deposition of lignin in phloem and xylem fiber cells of *Zinnia* *elegans* , while Blount et al. [38] observed that reduced levels of lignin correlated with a pronounced decrease in C4H activity in transgenic lines of tobacco. As noted herein, the increased activity of C4H (Figure 2) correlated with a pronounced increase in lignin content (Figure 3). These findings are of particular interest because a reduction in root growth has been considered one of the first effects of cinnamic acid derivatives that are is associated with the premature lignification of cell walls [15–17]. These citations and the current findings strengthen the notion of a possible influx of these compounds into the phenylpropanoid pathway, followed by increases of lignin monomers that solidify the cell wall and reduce root growth. Therefore, a similar function can be attributed to cinnamic acid itself. In fact, 0.1–1.0 mM cinnamic acid significantly increased the H lignin monomer and, as a result, altered both the sum (H+G+S) of the lignin monomers and the H: G:S ratios (Table 2).

**Figure 7:**
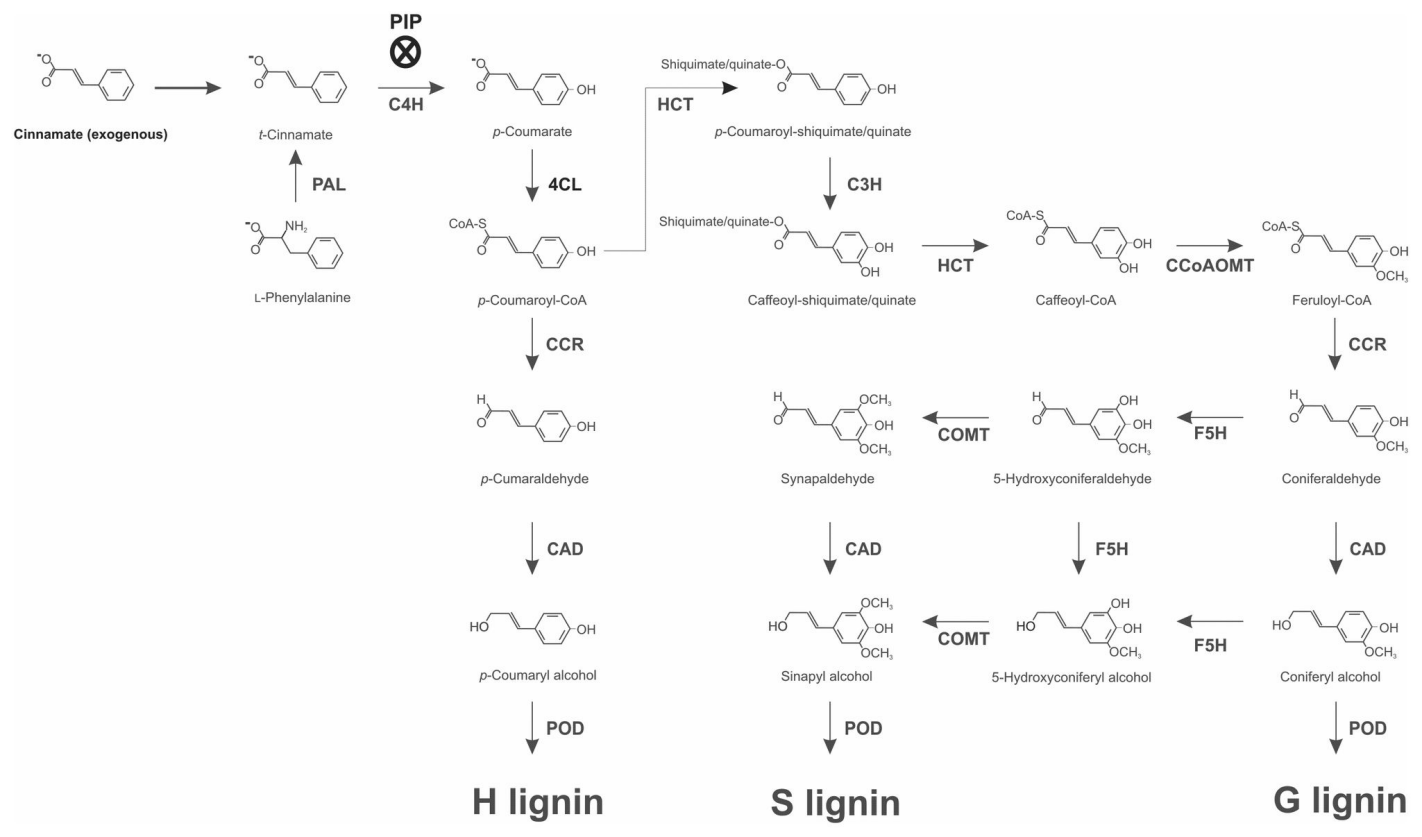
A simplified scheme for the synthesis of lignin and the proposed mode of action for cinnamic acid in soybean roots. PAL, phenylalanine ammonia-lyase; C4H, cinnamate 4-hydroxylase; 4CL, 4-coumarate: CoA ligase; CCR, cinnamoyl-CoA reductase; HCT, *p*-hydroxycinnamoyl-CoA: shikimate/quinate *p*-hydroxycinnamoyl transferase; C3H, *p*-coumarate 3-hydroxylase; CCoAOMT, caffeoyl-CoA 3-*O*-methyltransferase; CAD, cinnamyl alcohol dehydrogenase; F5H, ferulate 5-hydroxylase; COMT, caffeic acid 3-*O*-methyltransferase; POD, peroxidase. H, *p*-hydroxyphenyl; G, guaiacyl; S, syringyl. PIP is an inhibitor of C4H.

To confirm a supposed entry of exogenous cinnamic acid into the phenylpropanoid pathway, additional experiments were performed by growing soybean roots with PIP, a specific inhibitor of C4H. The findings reveal that alone or jointly with cinnamic acid, PIP reduced C4H activity (Figure 4), the lignin content (Figure 5) and H, G and S monomers (Figure 6) in comparison with the cinnamic acid treatment. These data indicate that roots grown under cinnamic acid treatments produce more lignin, while roots grown with PIP or cinnamic acid plus PIP synthesize less lignin. These results are in agreement with the fact that PIP acts on the entry point of cinnamic acid in the pathway [39]. Because PIP affects the C4H reaction, the exogenous allelochemical was blocked from accessing the phenylpropanoid pathway at this metabolic point. These results suggest that exogenously applied cinnamic acid can be channeled into the phenylpropanoid pathway and increase lignin production. In *Robinia* ( *Robinia* *pseudoacacia* ), labeled ferulic acid, which is another phenylpropanoid allelochemical, was incorporated into G and S lignin monomers, and these incorporations increased the cell-wall lignification [40]. In previous studies, we noted that cinnamic acid derivatives (i.e., ferulic, *p*-coumaric and caffeic acids) also increased the lignin content, inducing significant changes in the lignin monomer content [15–17]. Complementarily, Hrubcová et al. [41] also observed a decline in the IAA level in alfalfa suspension cultures subjected to 2-aminoindan-2-phosphonic acid (AIP), a known inhibitor of the phenylalanine ammonia-lyase (PAL), which is the first enzyme of the phenylpropanoid pathway that is the main route to the lignin production (Figure 7).

Our experimental findings have enabled us to provide a working model for the role of cinnamic acid as an allelochemical (Figure 7). In this model, exogenous cinnamic acid is channeled into the phenylpropanoid pathway and converted to *p*-coumarate through the C4H reaction and is, thereafter, converted to *p*-coumaryl-CoA → *p*-coumaraldehyde → *p*-coumaryl alcohol, which is a monolignol. In a subsequent reaction, this monolignol is polymerized into lignin in the cell wall. In this way, the cinnamic acid-induced inhibition of soybean root growth can be due to the excessive production of lignin, particularly the H monomer. Concomitantly, an increase in IAA oxidase activity reduces the endogenous IAA level associated with a decrease in root growth. In brief, the cinnamic acid-induced inhibition of soybean root growth can be due to the formation of a complex network that solidifies the cell wall and restricts the plant growth.
